# Inflammation: The Common Pathway of Stress-Related Diseases

**DOI:** 10.3389/fnhum.2017.00316

**Published:** 2017-06-20

**Authors:** Yun-Zi Liu, Yun-Xia Wang, Chun-Lei Jiang

**Affiliations:** Laboratory of Stress Medicine, Faculty of Psychology and Mental Health, Second Military Medical UniversityShanghai, China

**Keywords:** stress-related disease, inflammation, neuroimmunomodulation, neurotransmitter, cardiovascular disease, metabolic disease, depression, neurodegenerative disease

## Abstract

While modernization has dramatically increased lifespan, it has also witnessed that the nature of stress has changed dramatically. Chronic stress result failures of homeostasis thus lead to various diseases such as atherosclerosis, non-alcoholic fatty liver disease (NAFLD) and depression. However, while 75%–90% of human diseases is related to the activation of stress system, the common pathways between stress exposure and pathophysiological processes underlying disease is still debatable. Chronic inflammation is an essential component of chronic diseases. Additionally, accumulating evidence suggested that excessive inflammation plays critical roles in the pathophysiology of the stress-related diseases, yet the basis for this connection is not fully understood. Here we discuss the role of inflammation in stress-induced diseases and suggest a common pathway for stress-related diseases that is based on chronic mild inflammation. This framework highlights the fundamental impact of inflammation mechanisms and provides a new perspective on the prevention and treatment of stress-related diseases.

## Introduction

Stress is a state of threatened homeostasis provoked by a psychological, environmental, or physiological stressor. With rapid development of science and technology, as well as economy and strong social competition, the nature of stress has changed dramatically (Landsbergis, 2003). Stressful events engender multiple neurochemical, neurotransmitter and hormonal alterations by mainly activating the sympathetic nervous system (SNS) and the hypothalamic-pituitary-adrenal (HPA) axis. When stress stimuli are under control, the body responds to these in the physiological way. SNA and HPA axis are woken up to release chemical mediators to protect our body from stress. For instance, catecholamines are elevated to increase heart rate and blood pressure, which help us to fight or flight. This appropriate body reaction was called “allostasis” by Sterling and Eyer (1988). This state is beneficial to our survival and recovery. However, when stress stimuli are prolonged or over exaggerated, in another word, chronically increased allostasis lead to pathophysiology. In the last two decades, accumulating evidence indicated that severe or prolonged (chronic) stress resulted in increased risk for physical and psychiatric disorders, which is called stress-related disease. Stress is the common risk factor of 75%–90% diseases, including the diseases which cause the foremost morbidity and mortality. According to the former review, the most common stress-related diseases are cardiovascular diseases (CVD, i.e., hypertension and atherosclerosis), metabolic diseases (i.e., diabetes and non-alcoholic fatty liver disease, NAFLD), psychotic and neurodegenerative disorders (i.e., depression, Alzheimer’s disease, AD and Parkinson’s disease, PD), cancer (Cohen et al., 2007).

The traditional standpoint of mechanisms linking stress and disease has focused on the classical stress systems—the HPA axis and SNS. However, alterations in HPA axis and SNS mainly have indirect effects on target systems; thus the mechanisms link stress to stress-related diseases, and are still under debate. Recently, inflammation as a new and promising biological mechanism is proposed (Rohleder, 2014). Accumulating literatures showed that excessive inflammation directly contribute to pathophysiology of stress-related diseases. In this review article, the search terms were combinations of the following (literatures were selected from PubMed): stress (“social stress” or “psychosocial stress” or “psychophysiological stress” or “mental stress”), disease (“CVD” or “metabolic diseases” or “psychotic and neurodegenerative disorders” or “cancer”), and inflammation (“Inflammatory” or “cytokines”). We make a brief summary of stress and inflammation in the field of stress-related diseases. On the basis of these reports, we further hypothesize that inflammation may be one of the common pathways of stress-related diseases.

## Stress and Inflammation

Large bodies of evidence indicate that stress can activate inflammatory response in brain as well as peripherally (Rohleder, 2014; Calcia et al., 2016).

There exists communication between the neuroendocrine and immune systems (Jiang et al., 1998; Quan and Banks, 2007). Stress activates the HPA axis through the hypothalamic secretion of corticotropin-releasing hormone (CRH), which normally suppresses immune responses through the release of glucocorticoids (GCs) from the adrenals. GCs are one of the major stress hormones released during stress response that are well known for their immunosuppressive and anti-inflammatory properties. Studies during the 1970s and 1980s revealed that GCs inhibited lymphocyte proliferation and cytotoxicity. Further, GCs reduce the expression of several pro-inflammatory cytokines (e.g., tumor necrosis factor α (TNF-α), interleukin-6 (IL-6)) and enhance the expression of anti-inflammatory cytokines (e.g., IL-10, TNF-β; Sorrells et al., 2009). However, recent researchers have proved that GCs also have pro-inflammatory impact on immune system (Elenkov, 2008). Rats with higher basal plasma corticosterone levels have more accumulation of PGE_2_ whereas showing less anti-inflammatory factors after acute stress (Pérez-Nievas et al., 2007). GCs enhance the expression and function of inflammasome NLRP3, promoting the secretion of IL-1β in response to ATP. Inflammasomes are cytoplasmic multi-protein complexes that sense exogenous and endogenous danger signals and cleave pro-inflammatory cytokines into mature cytokines such as IL-1β and IL-18. This work demonstrates the proinflammatory role for GCs, enhancing the activation of the innate immune system in response to danger signals (Busillo et al., 2011). Circulating pro-inflammatory factors such as IL-1, IL-6 and TNFα directly stimulate the pituitary-adrenal axis, resulting in increased serum levels of adrenocorticotropic hormone (ACTH) and GCs, which in turn inhibit the production of these pro-inflammatory factors (Alley et al., 2006; Danese et al., 2007; Steptoe et al., 2007; Miller et al., 2008). The interaction of immune system and HPA axis form the endocrine negative feedback loops. However, when cytokine is over-stimulated in some diseases, these negative feedback loops could be weakened by reduced cytoplasmic GC-receptor (GR) level and decreased expression of GR driven anti-inflammatory genes, thus leading to GC low-responsiveness (Sterling and Eyer, 1988). Besides GCs, the SNS and its main neurotransmitter, norepinephrine (NE) and neuropeptide Y (NPY), could regulate the immune and inflammatory function. NE promoted the secretion of inflammatory factors by increasing the phosphorylation of mitogen-activated protein kinases (MAPKs) through an α receptor-dependent pathway and NPY could elicit transforming growth factor-β (TGF-β) and TNFα production in macrophage-like cell line RAW264.7 via Y1 receptor (Bellinger et al., 2008; Zhou et al., 2008; Huang et al., 2012).

Both pro-inflammatory and anti-inflammatory mechanisms depend on the type and intensity of stressors. Acute stressors seem to enhance immune function, whereas chronic stressors are suppressive. Intense stressors over-activate the immune system, leading to the imbalance of inflammation and anti-inflammation. Reports from different labs have confirmed pro-inflammation induced by stress, including C-reactive protein (CRP), IL-6, TNFα, IL-1β and the transcription factor of “nuclear factor kappa B (NF-κB)” (Miller et al., 2009).

In addition to peripheral inflammation, central inflammation namely neuroinflammation, has also been found in stress condition (García-Bueno et al., 2008; Munhoz et al., 2008). Elevated pro-inflammatory cytokines, increased microglia activation and accumulation of peripherally-derived monocytes and macrophages were detected in the brain with psychological stress exposure (Johnson et al., 2005). As the brain-resident macrophages, microglia was considered to be the major pro-inflammatory cytokine source. Stress-elicited potentiate microglial activation is via both direct and indirect mechanisms. Microglia express both GC and mineralocorticoid receptors, thus microglia are likely to have direct response to corticosterone peak (Calcia et al., 2016). In addition, GC receptors also are highly present in the hippocampus and prefrontal cortex, so stress-induced corticosterone may have indirect effects on microglia. Besides this, a recent research display that CNS innate immune system can respond to an acute stressor, thereby releasing the danger signal high mobility group box-1 (HMGB-1) in the brain to prime microglia by acting on the NLRP3 inflammasome, in preparation for IL-1β secretion (Weber et al., 2015). Activated microglia display hypertrophic branch morphology with an enlarged soma and produce an exaggerated cytokine to recruit peripheral monocytes. Increased brain macrophages and circulating monocytes, contribute to elevated levels of pro-inflammatory cytokine production (i.e., IL-1β, TNFα, IL-6) in the brain (Wohleb and Delpech, 2016).

In common, over-activated immune system, increased activity through SNS pathways, and reduced GCs responsiveness may work tandemly in the activation of inflammatory responses during stress. GCs, catecholamines, cytokines and other mediators released by stress are thought to be the main mediators in stress-induced pro-inflammatory effect.

## Inflammation and Diseases

Classically, inflammation is classically known as the crucial response to microbe invasion or tissue injury to keep maintenance of tissue homeostasis. In recent years, our knowledge of the inflammation role is greatly enlarged. Inflammatory pathway has been recognized as a pivotal molecular basis in the pathogenesis of many chronic diseases. By far, increasing literatures have shown that excessive inflammation play critical roles in the progression, and/or onset of stress-related diseases. There has been a growing number of evidence supporting that inflammatory response constitutes the “common soil” of the multifactorial diseases, including cardiovascular and metabolic diseases, psychotic neurodegenerative disorders and cancer (Scrivo et al., 2011).

## Stress, Inflammation and Diseases

Accumulating researches suggested that excessive inflammation plays critical roles in relationship between stress and stress-related diseases. Although stress and inflammation, or inflammation and diseases have been widely and nicely discussed, there are few literatures concerned of all these three factors (stress, inflammation and disease). In this part, we will discuss inflammation in different stress-related diseases and explore the inside mechanism (Table 1).

**Table 1:** Stress substance that link stress and various diseases.

Stress substance	Stress-related diseases	References
**Cardiovascular diseases**
Vasopressin	Hypertension	Szczepanska-Sadowska et al. (2010)
NE	Hypertension	Seidman and Standring (2010)
IL-6	Atherosclerosis	Nadrowski et al. (2016)
CRP	Atherosclerosis	Tsirpanlis (2005), Nadrowski et al. (2016)
**Metabolic diseases**
GC	Insulin resistance.	Mulder et al. (2005)
NE	Insulin resistance, Dyslipidemia	Marangou et al. (1988)
**Psychotic and neurodegenerative disorders**
IL-6	Depression	Henry et al. (2008)
NLRP3 inflammasome	Depression	Zhang et al. (2014)
PGs and PAF	Parkinson’s disease	Busillo et al. (2011)
**Cancer**
β-adrenergic signaling	Pancreatic cancer, acute lymphoblastic leukemia, breast cancer	Lamkin et al. (2012), Kim-Fuchs et al. (2014), Qin et al. (2015)
Catecholamines	Breast cancer	Lamkin et al. (2015)
IL-6	Lung cancer, pancreatic cancer, hepatocellular carcinoma	Grivennikov et al. (2009), Brenner et al. (2017), Lin et al. (2017)

*NE, Norepinephrine; GC, glucocorticoid; PGs, prostaglandins; PAF, platelet activating factor*.

### Stress, Inflammation and CVD

CVD was considered to be a leading cause of death worldwide. Large bodies of clinical trial pointed out that chronic stress, whether early life stress (Su et al., 2015) or adult stress has long been linked to increased coronary heart disease (CHD) risk. Childhood adversity especially severe physical and sexual abuse in childhood was found to strongly relate to higher morbidity of cardiovascular events in women (Rich-Edwards et al., 2012; Thurston et al., 2014). Children who are less expressive and cohesive in their original family exhibited more problematic cardiovascular risk factor profiles (Bleil et al., 2013). Those who experienced more family disruption events or early life family conflict had greater mean intima-media thickness (IMT), a subclinical marker of CVD risk (Bleil et al., 2013). In adulthood, work-related stressors such as low-income, high job demands combined with low control, shift work and workplace conflicts were mostly reported to be correlated to higher CVD risk (Bleil et al., 2013). Besides that, poor sleep quality under stress, discrimination emotion stress, such as anger, hostility and aggressiveness were also involved in coronary artery disease (Kop, 2003). On the contrast, effective stress management including positive emotions, optimism and life satisfaction were proved to have protective roles for CVD (Bleil et al., 2013).

While the biological mechanisms of stress increasing CVD risk are not well-known, chronic low-grade inflammatory load may emerge as a possible link as it is both elevated by chronic stress and contributed to early process, progression and thrombotic complications of atherosclerosis. IL-6 and CRP, the two important biomarkers of systematic inflammation, are considered indicative and potentially predictive for atherosclerosis (Tsirpanlis, 2005; Nadrowski et al., 2016). Coincidently, these two inflammatory indicators were elevated in different types of life stress. For instance, severe levels of childhood abuse were associated with a more elevated acute stress-induced IL-6 response, possibly due to reduced methylation of the IL-6 promoter (Janusek et al., 2017). Adults who had greater childhood adversity was reported to have more depressive symptoms and elevated concentrations of CRP (Janusek et al., 2017). Recent studies have suggested that CRP and IL-6 are mechanisms by which early adversity may contribute to CVD (Ridker et al., 2002; Albert et al., 2006; Graham et al., 2006). Work-related stressors have also been mentioned to correlate with elevated CRP and IL-6 (von Känel et al., 2008). In a recent study applied in black and white men, greater stressor-evoked reduction in high-frequency heart rate variability (HF-HRV) and were correlated with higher CRP and IL-6. In animal stress models (social isolation, social disruption, cold stress, severe chronic unpredictable stress), increased plaque size, elevated serum IL-6, NPY levels were observed. However, when single supplied with GC after Adrenalectomy, plaque size and serum inflammatory factors were decreased or did not change. This suggested that the possible mechanisms of stress-related inflammation in CVD may include SNS-mediated increases in NE and NPY. Noisy communities as life stressor induces significant increase in urine epinephrine and NE leading to hypertension (Seidman and Standring, 2010). NE promoted the production of inflammatory factors by facilitating the phosphorylation of MAPKs through activation of NE α receptor (Huang et al., 2012). NPY could elicit TGF-β1 and TNFα production in macrophage-like cell line RAW264.7 via Y1 receptor (von Känel et al., 2008). NPY could also directly activate the HMGB1 release and cytoplasmic translocation from the macrophage (Zhou et al., 2013). Inflammation has also been shown to correlate with endothelial dysfunction and relate to the renin-angiotensin system (Li et al., 2012).

Overall, the possible mechanism could be summarized as follows. Stress may activate through SNS system to release NE and NPY, these two stress hormones further facilitate the phosphorylation of MAPKs or HMGB1 release, therefore inducing systematic inflammation (IL-6, CRP) to promote or accelerate CVD development. Anti-inflammatory drugs may have synergistic effect with conventional antihypertensive drugs on the prevention and treatment of stress-related CVD.

### Stress, Inflammation and Metabolic Disease

Stressful events could motivate unhealthy food choices (Kuo et al., 2008). These unhealthy foods are frequently associated with morbid obesity, type 2 diabetes mellitus, metabolic syndrome and NAFLD (Mikolajczyk et al., 2009). Stress enhances both post-meal peaks of triglycerides and delays lipids clearance (Kiecolt-Glaser, 2010). As shown in Hoorn’s study, stressful life events, which indicate chronic psychological stress, are associated with higher prevalence of undetected type 2 diabetes (Mooy et al., 2000). A recent prospective study supported this view, and provided further evidence (Cosgrove et al., 2012). Furthermore, effective stress management training or mindfulness-based stress reduction training has been proved to have clinically significant benefits on patients with type 2 diabetes. On the contrary, highly anxious patients did not obtain more improvement from the training (Rosenzweig et al., 2007).

Insulin resistance frequently develops during acute or chronic stress (Tsuneki et al., 2013). Insufficient insulin secretion to compensate for insulin resistance is also the characteristic of Type 2 diabetes. Insulin resistance, visceral obesity, dyslipidemia, type 2 diabetes mellitus and metabolic syndrome are key risk factors in the development and progression of NAFLD. At the intersection of metabolism and immunity, inflammation may be an important link between stress and metabolic disease. Intense stress over-activates the immune system, leading to the imbalance between inflammation and anti-inflammation. The activated stress pathways can initiate or exacerbate inflammation and culminate in hepatocyte cell death and liver damage by apoptosis (Gentile et al., 2011). IL-1 family members might be involved in controlling insulin resistance and metabolic inflammation in various obesity-associated disorders (Kamari et al., 2011; Tilg and Moschen, 2011; Tack et al., 2012). It is reported that the modulator of IL-1, NLRP6 and NLRP3 inflammasomes negatively regulate NAFLD/NASH progression, as well as multiple aspects of metabolic syndrome (Zhu et al., 2006). Inflammatory transcriptor NF-κB and JNK activator protein-1 (AP-1) emerged as a central metabolic regulator (Wellen and Hotamisligil, 2005). Enhanced hepatic NF-κB activity was observed in high fat fed-mice (Day, 2006). NAFLD is regularly associated with lipometabolic disorders and inflammatory reactions, especially in the nonalcoholic steatohepatitis (NASH) stage (Liu et al., 2012). Chronic, low-grade inflammatory process is also the characteristic of diabetes. The “two-hit” hypothesis for the pathogenesis of NAFLD implicates inflammation as the link between steatosis and steatohepatitis. Inflammatory stress may aggravate the progression of NAFLD by increasing cholesterol influx and reducing cholesterol efflux especially during the second-hit stage of NAFLD (Ma et al., 2008).

Metabolism-controlling stress hormones, especially GCs and NE could exert anti-insulin effects, and in the long run induce insulin resistance. GC receptor antagonist RU486 and adrenalectomy reduce the occurrence of insulin resistance. High concentration of NE in plasma could raise fasting glucose and reduce glucose tolerance, possibly mediated by lipolysis and increased fatty acid concentrations (Marangou et al., 1988). Adrenergic receptor activation may directly affect the insulin signaling pathway or cellular glucose transport (Mulder et al., 2005). Additionally, GCs and NE could also regulate inflammation. In diabetes, elevated circulating levels of proinflammatory cytokines are originally thought to be the adipocytes themselves in response to obesity. However, an increasing number of evidence suggests that obesity results in increased number of macrophages and changes in the activation status of these cells. Therefore, adipose tissue macrophages produce a significant proportion of the inflammatory factors that are upregulated by obesity (Donath and Shoelson, 2011). Inflammatory cytokines produced by various cells such as Kupffer cells, macrophages, neutrophils, monocytes, adipocytes and hepatocytes, have critical roles in lipid metabolism and hepatic inflammation that promote liver damage. Antagonizing or inhibiting TNFα, significantly improved NAFLD and is currently tested in human NASH (chronic hepatic inflammation; Gastaldelli et al., 2009; Musso et al., 2009). Furthermore, TNFR1 ectodomain shedding could attenuate the progression from “simple steatosis” towards NASH (Aparicio-Vergara et al., 2013).

### Stress, Inflammation and Depression

Stressful experiences are fundamental in the provocation of major depression of disorder (MDD). HPA axis activation and hypercortisolemia often seen in depressed patients may represent increased stress hormones, CRH and ACTH secretion (Capuron et al., 2003). MAPK pathways have been proved to increase the activity of serotonin membrane transporters, the most important neurotransmitter associated with depression (Zhu et al., 2006).

Recently, the “cytokine hypothesis” or “macrophage theory” has been suggested in MDD. The main idea of inflammatory depression is the activation of the inflammatory immune response, particularly the synthesis of cytokines, which might influence neurochemicals and contribute to MDD (Smith, 1991). Stress can facilitate the development of depressive-like behavior by promoting inflammatory cytokine expression (Norman et al., 2010). Additionally, a new pathway—kynurenine pathway (KP) has attracted much more attention in cytokine hypothesis. Proinflammatory cytokines activate KP to affect tryptophan metabolism and produce neurotoxin, which either reduces serotonin synthesis or fastens the reuptake of serotonin (Miura et al., 2008).

Data from animal models and clinical patients prove the role of inflammation in depression. Exposure to inflammatory cytokines such as TNFα, IFNα and IL-1β or cytokine inducers such as LPS or vaccination has been shown to lead to marked behavioral alterations in human and rodent. Elevated inflammatory mediators such as cytokines and their soluble receptors, chemokines, acute phase proteins, adhesion molecules and prostaglandins (PGs) have also been found with depression in peripheral blood, CNS and cerebrospinal fluid (CSF; Miller et al., 2009; Dowlati et al., 2010; Norman et al., 2010; Raison et al., 2010). We use chronic stress to establish depression model. Four-week chronic stress exposure significantly upregulates the inflammatory cytokines such as TNFα, IL-18, IL-1β and inflammatory inducible NOS (iNOS) expression (Peng et al., 2012). Accompanying the upregulation of proinflammatory cytokines, depressive-like behaviors were established. In contrast, blocking iNOS or inflammatory cytokines with 1400W (Peng et al., 2012) or minocycline could abrogate the depressive-like behavior induced by stress (Peng et al., 2012). In fact, some clinical antidepressants really have the role of anti-inflammation. Antidepressant drug and nonsteroidal anti-inflammatory drugs (NSAIDs) like minocycline, decrease blood levels of IL-6, attenuate microglial activation and central cytokine secretion and behavioral changes (Henry et al., 2008).

Inflammasomes are multi-molecular platforms, driving the maturation and secretion of pro-inflammatory factors IL-1β and IL-18 to take part in innate immune defenses (Schroder and Tschopp, 2010). We found that NLRP3 inflammasome is involved in LPS-induced mice depressive-like behaviors (Zhang et al., 2014). Recent research showed protective effect of caspase-1 inhibition on brain function, and gut microbiota induced depressive- and anxiety-like behaviors (Wong et al., 2016).

### Stress, Inflammation and Neurodegenerative Diseases

The role of stress and inflammation are being recognized in neurodegenerative disease. AD and PD are the two most common neurodegenerative diseases. Extracellular amyloid β protein (Aβ) accumulation is currently seen as a key step in the pathogenesis of AD. PD is characterized by progressive loss of nigrostriatal dopaminergic (DA) neurons and depletion of dopamine in the striatum, which lead to pathological and clinical abnormalities. The potential etiology and molecular mechanisms underlying the pathogenesis of AD and PD remains unknown and have not been completely elucidated. However, some progress has been made in identifying the risk factors. During the last two to three decades, increasing evidence from animal and clinical studies has implicated stress and neuroinflammation as risk factors and may play a fundamental part in the pathogenesis of AD and PD.

Epidemiological, clinical studies and animal model of AD suggest that stress and inflammation interact with processing and deposit of Aβ, contributing to the pathogenesis of AD (Kunjathoor et al., 2004). Hypercortisolemia is one of the features found in patients diagnosed of AD. An array of elevated inflammatory mediators including TNFα, IL-1, PGE_2_, NF-κB, COX-2 and MCP-1 has been detected from patients with AD (Wyss-Coray, 2006; Comi et al., 2010) and correlated with the amount of Aβ and the severity of AD pathogenesis (Hoshino et al., 2009; Chen et al., 2012). Researchers also observed increased cytokines such as TNFα, IL-1β and IFN in the substantianigra of PD patients (Nagatsu and Sawada, 2005). Activation of the systemic innate immune system by infection may participate in the early stages of AD pathogenesis (Perry et al., 2007). Neuroinflammation induces degenerative changes in the DA system, which lowers the set point toward neuronal dysfunction and degeneration (Morand and Leech, 1999). Proinflammatory lipid mediators include PGs and platelet activating factor, together with cytokines may significantly affect the progressive neurodegeneration in PD (Busillo et al., 2011). Mice with microglial activation-induced oxidative stress and inflammation, and nigrostriatal DA neuronal damage have been used to serve as an experimental model of PD. Stress exposure increased neuroinflammation in AD and is characterized by astrogliosis, increased inflammatory gene expression and lipid peroxidation (Perez Nievas et al., 2011). It has been confirmed with the changes in glial cells surrounding the senile plaques. Genetic research demonstrates that inherited variations in inflammatory response mechanisms may influence AD pathogenesis (Grimaldi et al., 2000; Nicoll et al., 2000). In contrast, anti-inflammatory agents such as NSAIDs and antioxidant therapy might protect against the development of AD. Long-term use of NSAIDs, inhibitors of COX, suppression of neuroinflammation by glial inhibitors, delays the initiation and reduces the risk of AD (Tsukuda et al., 2009; Chen et al., 2012). In consistent with epidemiology, nicotine was proved to have a neuroprotective effect on DA neurons by means of an anti-inflammatory mechanism mediated by the regulation of microglial activation (Park et al., 2007). Therefore, new potent neuroprotective therapies for PD might be taken into account by focusing on critical inflammatory mechanisms, such as cytokine-induced neurotoxicity (Morand and Leech, 1999). A variety of preclinical studies have corroborated the therapeutic potential of targeting cholinergic anti-inflammatory pathway (Bencherif et al., 2011).

### Stress, Inflammation and Cancer

Chronic stress has been demonstrated to account for a place in physiological and pathological disease outcomes, including several types of cancers (Krizanova et al., 2016). Chronic stress is thought to correlate with the etiology of tumor growth, progression and metastasis (Thaker et al., 2006). In a clinical study of breast cancer patients 3 years post-treatment, elevated levels of stress-inducible acute phase proteins correlated with an increase in morbidity and mortality in the experimental cohort (Pierce et al., 2009). Furthermore, animal experiment by using daily exposure to a novel environment to explore the effect of stress on the growth rate of SC115 carcinoma showed that social housing condition and novelty stress may lead to various impacts on the growth rate of tumor in mice (Kerr et al., 1999). Metastasis is the main cause of death in cancer patients. Researchers demonstrated that chronic stress accelerates liver metastasis of colorectal cancer breast cancer and prostate cancer metastasis (Barbieri et al., 2015; Zhao et al., 2015; Wong et al., 2016).

Classic stress signal, β-adrenergic signaling activation is considered as the main cause of pancreatic cancer, acute lymphoblastic leukemia, breast cancer progression and invasion (Lamkin et al., 2012; Kim-Fuchs et al., 2014; Qin et al., 2015). These effects were showed to have relevance with increased expression of invasion genes in tumor cells. Pharmacological β-adrenergic blockade antagonist could reverse the observed effects of chronic stress on cancer progression. Furthermore, activation of β-adrenergic signaling by βAR agonists reduces the deformability of highly metastatic human breast cancer cells, ovarian, prostate, melanoma and leukemia cells, which depends on the actin cytoskeleton and myosin II activity. These changes in cell deformability can be prevented by pharmacological β-blockade or genetic knockout of the β2-adrenergic receptor (β2-AR; Kim et al., 2016). Besides βAR, catecholamines also signal α-adrenergic receptors. Inversely, α2-adrenergic signaling was proved can inhibit sympathetic catecholamine release through an autoreceptor mechanism. Selective α2-adrenergic blockade mimics the accelerating effect of chronic stress on breast cancer progression (Lamkin et al., 2015).

The β2-ARs are expressed on multiple cell types involved in immunoregulation, including not only immune cells (Theron et al., 2013; Padro and Sanders, 2014), but also non-immune cells with a bystander role in the immune response (e.g., glia cells, fibroblasts, endothelial cells, etc.; Mantyh et al., 1995; Johnson, 2006). Stress-induced epinephrine binds to β2-ARs, and then results in the activation of p38 MAPK, which in turn enhances NF-κB DNA binding and cytokines and chemokines expression (Kolmus et al., 2015). More recently, stress-mediated immune modulation of cytokines including TNF-α, TGF-β, IL-1 and IL-6 have been suggested as indictors of cancer progression, metastasis and recurrence. Additionally, in some cancers (e.g., colon, renal cell, lung and breast) secretion of these same cytokines by tumor cells helps drive and sustain pro-tumorigenic inflammatory loops (Angelo et al., 2002; Gao et al., 2007). Among several cytokines, IL-6 is the most studied pro-inflammatory factor in tumor. Circulating levels of IL-6 have been reported as forecast cytokine of survival and metastasis in human cancers (Chung and Chang, 2003; Salgado et al., 2003; Pierce et al., 2009). Several studies revealed that high serum concentration of IL-6 is a prognostic indicator of poor outcome in cancer patients with diverse tumor types including gastric, pancreatic, melanoma, breast, colorectal, myeloma and lung cancer (Heikkilä et al., 2008; Lippitz, 2013). A higher lung cancer risk for participants with elevated concentrations of IL-6 was observed in recent clinical trial (Brenner et al., 2017). In animal studies, IL-6 trans-signaling is linked to tumor development in inflammation-induced colorectal and pancreatic cancer (Grivennikov et al., 2009; Rose-John, 2012). Moreover, evidence that disruption of IL-6 trans-signaling delays growth in established murine tumors demonstrates that IL-6 activities are important during neoplastic progression (Grivennikov et al., 2009; Rose-John, 2012). IL-6 trans-signaling-dependent activation of STAT3 can drive cancer progression through the transcription of target genes including the cell cycle regulator cyclin D1, the proto-oncogene c-myc, transcriptional regulators such as JunB, cFos, C/EBPβ and C/EBPδ, and metabolic regulators such as mTORC1 (Hirano et al., 2000; Thiem et al., 2013). IL-6 blockade would change immunological environment and reinforce the effectiveness of anti-programmed death-1-ligand 1 (anti-PD-L1) therapy, therefore evoking significant tumor suppression activity in pancreatic ductal adenocarcinoma (Mace et al., 2016); additionally, neutralization of IL-6 abrogated hepatocellular carcinoma (HCC) progression and myeloid-derived suppressive cells (MDSC) accumulation in Rarres2^−/−^ mice (Lin et al., 2017). Taken together, evidence linking stress to cancer progression and inflammation provide penetration into the magnitude of modulation of cancer-related cytokines (e.g., IL-6) that appear to alleviate the effects of stress on cancer.

## Conclusion

**Figure 1:**
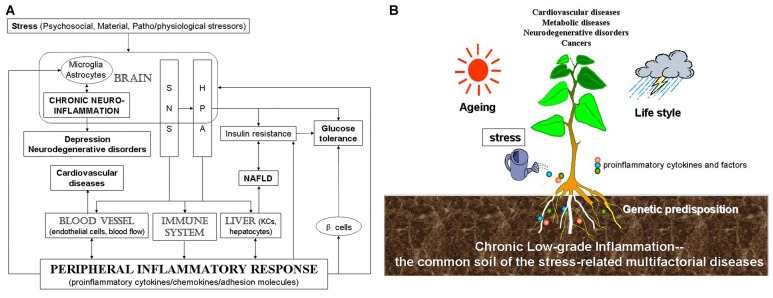
Scheme for the relationship among stress, inflammation and stress-related diseases.** (A)** Stress, including psychosocial, material, patho/physiological stressors, induces chronic CNS and peripheral inflammation, which is then related to stress-related diseases. **(B)** Stress-induced chronic low-grade inflammation might be the common soil of stress-related diseases. Multifactorial factors, including genetic predisposition, aging and life style and so on, act on stress-related diseases. Stress-induced inflammatory response represents the common soil of a wide variety of the chronic multifactorial diseases.

In summary, through disturbing the balance of immune system, stress induces inflammation peripherally and centrally. This imbalance leads to diversified stress-related diseases. Although there might be various different triggering events, they appear to converge on inflammation. In this review article, we provide evidence that stress induces or worsens CVD, NAFLD, depression, neurodegenerative disease and cancer through peripheral inflammation as well as neuroinflammation. Stress engenders central microglia and astrocytes, blood vessel, immune system and liver by mainly activating SNS and the HPA axis (Figure 1A). Therefore, we suggested that inflammation may be the common pathway for stress-related diseases, which may act as a factor that contributes disease progression or may occur very early during the development of the disease. Figure 1B shows that multifactorial factors, including genetic predisposition, aging and life style, act on stress-related diseases and that stress-induced chronic low-grade inflammation is the common soil of a wide variety of the chronic diseases.

### Limitations

Stress-induced inflammation described here may be relevant to understand the common mechanisms of stress-related diseases. However, quite a few unanswered questions still need to be further discussed. For instance, besides inflammation, is there the crosstalk among inflammation and other related pathways such as cell stress? Is there the specific cell or pathway for the specific stress-related disease? Can anti-inflammatory specifically affect neuroinflammation without modulating periphery immunity for CNS disease? More crucially, to reach clinical application, anti-inflammatory therapies will need to accurately target on specific cells and pathways in CNS, which are fundamentally important in human disease pathogenesis. All these limitations could be the next research key point. Breaking through these barriers would make great progress on the treatment of stress-related diseases.

### Future Directions

Overall, one thing is clear at present time. To improve stress condition, reduction of psychological and physical stress should be put on the agenda of the patients with a wide variety of the chronic multifactorial stress-related diseases. Furthermore, interventions targeting stress risk factors, especially stress-induced inflammation, would be beneficial for the treatment of diseases (mainly aiming at specific inflammatory factors), especially for disease prevention among the highly stressful people (mainly anti-inflammation non-specially).

## Author Contributions

C-LJ designed the work and edited the manuscript. Y-ZL and Y-XW did the literature research and prepared the manuscript. All authors read and approved the manuscript.

## Conflict of Interest Statement

The authors declare that the research was conducted in the absence of any commercial or financial relationships that could be construed as a potential conflict of interest.
